# Efficiency of Emission Control Measures on Particulate Matter-Related Health Impacts and Economic Cost during the 2014 Asia-Pacific Economic Cooperation Meeting in Beijing

**DOI:** 10.3390/ijerph14010019

**Published:** 2016-12-28

**Authors:** Qichen Liu, Jing Huang, Bin Guo, Xinbiao Guo

**Affiliations:** Department of Occupational and Environmental Health Sciences, School of Public Health, Peking University, Beijing 100191, China; liuqichen@bjmu.edu.cn (Q.L.); jing_huang@bjmu.edu.cn (J.H.); 15210594187@163.com (B.G.)

**Keywords:** particulate matter, PM_2.5_, PM_10_, Asia-Pacific Economic Cooperation meeting, emission control measures, health economic cost

## Abstract

*Background*: The Asia-Pacific Economic Cooperation (APEC) meeting was held from 5 November to 11 November 2014 in Beijing, and comprehensive emission control measures were implemented. The efficiency of these measures on particulate matter-related health impacts and economic cost need to be evaluated. *Methods*: The influences of emission control measures during APEC on particulate matter were evaluated, and health economic effects were assessed. *Results*: Average concentrations of PM_2.5_ and PM_10_ during APEC were reduced by 57.0%, and 50.6% respectively, compared with pre-APEC period. However, the concentrations of particulate matter rebounded after APEC. Compared with the pre-APEC and post-APEC periods, the estimated number of deaths caused by non-accidental, cardiovascular and respiratory diseases that could be attributed to PM_2.5_ and PM_10_ during the APEC were the lowest. The economic cost associated with mortality caused by PM_2.5_ and PM_10_ during the APEC were reduced by (61.3% and 66.6%) and (50.3% and 60.8%) respectively, compared with pre-APEC and post-APEC. *Conclusions*: The emission control measures were effective in improving short term air quality and reducing health risks and medical expenses during 2014 APEC, but more efforts is needed for long term and continuous air quality improvement and health protection.

## 1. Introduction

The rapid industrialization and urbanization in China have led to a drastic increase in energy consumption and pollutant emissions, especially in mega-cities. These emissions have exacerbated environment pollution problems, resulting in hazy weather and health concerns. Ambient air pollution ranks fourth among risk factors that contribute to the largest number of attributable disability-adjusted life-years (DALYs) in China [1]. In addition, air pollution caused 5.5 million deaths and 141.5 million global DALYs in 2013 [2].

Previous studies estimated that more than 19.2 million people (98% of Beijing population) are exposed to harmful level of long term PM_2.5_ pollution, and the population-weighted mean PM_2.5_ exceeded China’s daily PM_2.5_ standard of 75 μg/m^3^ [3].

In Beijing, the capital of China, remarkable problems of traffic and particulate air pollution have emerged, especially the fine particulate matter (PM_2.5_). The 2014 Asia-Pacific Economic Cooperation (APEC) meeting was held between 5 November and 11 November in Beijing. To achieve good air quality and ease traffic pressure, the Chinese government implemented comprehensive air pollution control measures from 3 November 2014 to 12 November 2014. These measures included restricting personal vehicles using an even-odd license plate system, forbidding the local trucks within the Sixth Ring Road between 6:00 a.m. to 24:00 p.m., and not permitting the non-local trucks into the city. In addition to mobile sources of particulate matter described above, fixed sources like power plants were also strictly controlled during this period in Beijing, including restricting the production activities of power plants and other factories, controlling construction activities, taking dust control measures, and so on. The surrounding regions including Tianjin, Hebei province, Shanxi province, Shandong province and Inner Mongolia also carried out mobile sources and fixed source control measures. 

It was possible that the concentrations of the particulate matter would decrease to a certain degree under such strict control measures, including a decrease in the corresponding health impacts and economic cost. In order to make a quantitative estimation of our hypothesis, we carried out this study. The objectives were summarized as follows: (1) Compare the concentrations of PM_2.5_ and PM_10_ during, pre- and post-APEC; (2) Evaluate the health benefits of emission control measures during APEC; (3) Assess the health economic cost associated with particulate matter pollution during, pre- and post-APEC.

## 2. Materials and Methods

### 2.1. Data Collection

In this study, concentrations of particulate matter (PM_2.5_ and PM_10_) were obtained from Beijing Municipal Environmental Monitoring Center, updated each hour [4]. In addition to the concentrations of particulate matter, meteorological conditions including environmental temperature, relative humidity, wind speed and direction, as well as the precipitation situation were recorded from China Meteorological Science Data Sharing Service Network [5]. We collected hourly concentrations of particulate matter and meteorological conditions from 20 October 2014 to 1 December 2014. To rule out the impacts of emission control measures on non-APEC days, several days that were in close proximity to APEC days were not entered into analysis, therefore we chose the dates between 20 October and 1 November as the pre-APEC period, the dates between 3 November and 12 November as the during-APEC period, and the dates between 19 November and 30 November as the post-APEC period.

### 2.2. Site Information of Air Pollutants Concentrations

**Figure 1:**
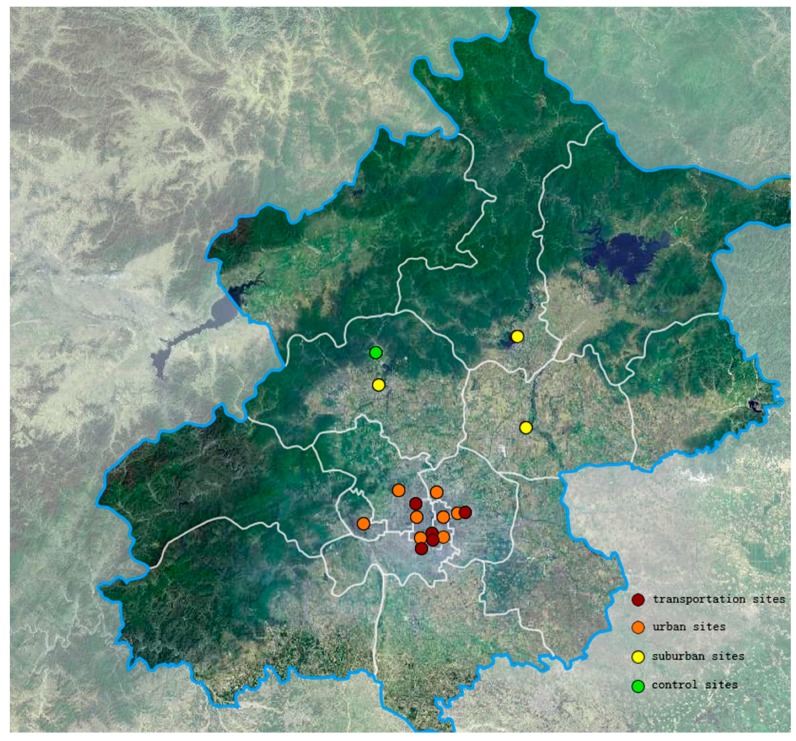
Distribution of monitoring sites.

There were four types of air pollution monitoring sites in Beijing, including urban, suburban, traffic and control sites. We collected data from all these types of sites. Among these sites, urban monitoring sites are Dongsi, Tiantan, Guanyuan, Wanshouxigong, Olympic Sports Center, Agriculture Exhibition Center, Wanliu, and Guchen, which are mainly distributed in Dongcheng, Xicheng, Chaoyang, and Haidian districts. Suburban monitoring sites include Shunyi Xincheng, Changping Town and Huairou Town, which are relatively distant from the urban area. The traffic sites are dispersed at main roads, including Qianmen East Street, Yongdingmen Inner Street, Xizhimen North Street, West Road of South 3rd Ring and North Road of East 4th Ring Road, respectively. The control site is Dingling seated in Changping district. It is far away from pollution sources and may reflect the background level of air pollution in Beijing (Figure 1). The monitoring sites above cover the study region and thus are considered to give full insights into the comprehensive status of air pollution in Beijing. 

### 2.3. Choice of Health Endpoints

When choosing health endpoints of particulate matter pollution, we considered the following principles: (1) the feasibility of data, preferably the heath points provided by reliable health statistics, such as those based on International Classification of Diseases (ICD-10); (2) health endpoints, which have data of exposure–response relationships. According to these principles, we selected death as health endpoints of particulate matter pollution, and deaths from non-accidental, cardiovascular and respiratory diseases were taken into consideration. 

### 2.4. Exposure-Response Function and Coefficients Selection

The incidence of death caused by particulate matter is a small probability event in the context of a population, which accords with Poisson distribution. Previous studies indicated that there was a roughly linear exposure-response relationship between particulate matter and related health endpoints [6,7,8]. Thus our study utilized a linear exposure–response model (Equation (1)), which is accepted and widely used by many researchers to evaluate the health economic effects [9,10,11]. The numbers of the incidence of health endpoints caused by particulate matter was calculated as follows (Equation (2)).

E = E_0_ × exp(β × (C − C_0_)) (1)

N = P × (E − E_0_) = P × E × (1 − 1/exp(β × (C − C_0_))) (2)

In both Equations (1) and (2), C is the actual concentration of particulate matter (μg/m^3^) and C_0_ is the threshold concentration of particulate with observed harmful health effects. We take the average year guiding values set by WHO as C_0_ in our study, with PM_2.5_ at 10 μg/m^3^ and PM_10_ at 20 μg/m^3^. E_0_ (%) and E (%) are the corresponding mortality at C_0_ and C respectively. P means the exposure population. According to “Beijing National Economic and Social Development Statistics Bulletin in 2014” released by Beijing municipal bureau of statistics, the residential population in Beijing were 21.516 million at the end of 2014. N represents the number of additional mortalities caused by specific particulate matter and β means the coefficient of exposure-response function of specific health endpoints, which refers to the change in the concentrations of certain particulate matter per 1 μg/m^3^, the corresponding change in the incidence of health endpoints.

In order to better evaluate the health effects of particulate matter, we preference integrated results from domestic studies as β [12], and annual mortality were mainly from Beijing statistical yearbook.

We supposed that the size of the baseline population in Beijing changed slightly during the investigation period and therefore the person-time units in the denominators of the mortality rate could remain constant; this allowed us to compare the average daily mortality counts before, during and after the APEC. 

In addition, we explored the health effects of particulate matter by AirQ+ software developed by the World Health Organization to compare and test our results. 

### 2.5. Evaluation Model of Health Economic Cost of Particulate Matter

Based on the method of “willing to pay”, society is willing to pay for the cost in order to prevent premature death caused by environmental pollution, called value of statistical life (VOSL) [13]. VOSL in statistical significance is known as the value which society would like to pay to reduce a certain risk of death or to prevent premature death. Compared with other methods evaluating economic cost, the VOSL method could reveal all of the losses of individual welfare caused by diseases or death, including the time cost, income loss and medical expense [14]. Hence, the VOSL was utilized to evaluate the health economic cost of excessive particulate matter concentration. Health economic cost caused by particulate matter pollution is equal to the number of the impaired multiplied by the value of statistical life; the evaluation model was as follows:

Cost = N × VOSL
(3)

In Equation (3), cost means the total value of health damage caused by particulate pollution; N represents the number of change in health endpoints; VOSL is unit value corresponding to the change in health endpoints.

Based on the surveys that investigate the willingness to pay (WTP) for the health benefits of the exposed population from air pollution reduction in Beijing, and calculate the health economic loss of particulate pollution against the exposed pollution in Beijing, we knew that VOSL for health risk of air pollution was 108,582.4–217,002.0 dollars [15]. With this information, we could draw conclusions about health economic cost of particulate matter.

### 2.6. Statistical Analysis

We compared the daily concentrations of particulate matter in different periods of APEC and diverse types of monitoring sites by Kruskal-Wallis test—one of the non-parametric tests.

In order to explore the effects of emission control measures on particulate matter, we used linear mixed effect models while controlling for meteorological factors (temperature, relative humidity and wind speed) and other confounding factors (such as the monitoring date, hours of the day). Hourly concentrations of particulate matter were treated as dependent variables, while periods (pre-APEC, during-APEC and post-APEC) that indicated the effects of emission control measures were treated as independent variables. Correlations of repeated measures within a day were taken into consideration by including the date of concentration collection as a random effect term, and other independent variables were treated as fixed effect terms. 

We evaluated non-accidental, cardiovascular and respiratory disease deaths attributed to PM_2.5_ and PM_10_ exposure with the method of exposure-response function, and the health economic cost associated with particulate matter during APEC-days and non-APEC days by method of VOST as described above.

All statistical analyses were performed by SAS software for Windows (version 9.1; SAS Institute Inc., Cary, NC, USA) and the level of significance was defined as *p* < 0.05 (2-tailed).

## 3. Results

### 3.1. Comparisons of Particulate Matter Concentrations in Different Periods

We calculated the average, median, minimum, maximum and percentile number concentrations of particulate matters recorded by the monitoring sites in the study. As shown in Table 1, the average concentrations of PM_2.5_ were 55.91 ± 29.33 μg/m^3^ during APEC days, while the average concentrations of PM_2.5_ were 130.15 ± 87.18 μg/m^3^ and 145.42 ± 89.58 μg/m^3^ pre-APEC and post-APEC, respectively. The concentrations of PM_2.5_ on non-APEC days were significantly higher than those of the during-APEC period (χ^2^ = 10.193, *p* = 0.006). For PM_10_, the same trend and results were observed, but compared with the reduction rates of PM_2.5_ (57.0% for pre-APEC vs. 61.5% for post-APEC), the decrease degree of PM_10_ was slightly lower (50.6% for pre-APEC vs. 61.0% for post-APEC) (Table 1). 

When controlling for meteorological factors (temperature, relative humidity and wind speed) and other confounding factors, the emission control measures also showed great contribution in reducing the concentrations of particulate matter (Table 2). Specifically, the difference between post-APEC and during-PAEC is much larger than that between pre-APEC and during-APEC for both PM_2.5_ and PM_10_; heating supplied after the APEC may play an important role for the disparity. The difference of PM_2.5_ between pre-APEC and during-APEC is not significant, whereas the difference of PM_10_ is significant between the same stage, the difference of physical property between PM_2.5_ and PM_10_ could account for this discrepancy. PM_2.5_ is smaller in aerodynamic diameter and can suspend in the air for a long time, and is also hard to diffuse compared with PM_10_. Although emission control measures had been taken during-APEC, a portion of PM_2.5_ produced previously may still remain in the atmosphere, resulting no significant difference of PM_2.5_ between pre- and during-APEC.

The concentrations of PM_2.5_ and PM_10_ were not significantly different among diverse types of monitoring sites in total observation (Supplementary Materials Table S1). 

### 3.2. Evaluation of the Health Benefits of Emission Control Measures during APEC

We evaluated the average daily excessive mortality of pre-, during- and post-APEC periods. Based on the exposure-response coefficient of β and actual mortality cited from the Beijing statistical yearbook, health risk of particulate matter pollution could be clearly evaluated. According to Table 3, the average daily estimated number of non-accidental deaths caused by PM_2.5_ during the APEC was the least among the three periods, and approximately eight deaths might have been prevented per day during the APEC. The average daily cardiovascular disease deaths caused by PM_2.5_ pollution was 4.8 pre-APEC, and decreased to 1.8 during the APEC. However, the number increased to 5.3 in post-APEC period, even higher than the pre-APEC period. The variation of average daily respiratory disease deaths showed the same change trend as the cardiovascular disease deaths.

Similar to the results of the additional mortality caused by PM_2.5_ pollution, the least average daily non-accidental, cardiovascular and respiratory deaths caused by PM_10_ also appeared during the APEC period. However, the decline of additional deaths caused by PM_10_ were not as dramatic as that related to PM_2.5_, which were consistent with the results of the concentrations’ variations. 

The results were similar when using AirQ+ software; the detailed information was presented in Table S2.

### 3.3. Comparisons of the Economic Cost Associated with Particulate Matter Pollution during Different Periods

In line with the mortality and unit economic cost of health endpoints, economic cost of specific health endpoints could be analyzed. As seen in Table 4, the minimum economic cost of non-accidental deaths caused by PM_2.5_ per day was U.S. $541,912 during the APEC, reduced by 61.3% and 66.6% respectively compared with pre-APEC and post-APEC, which was 0.06% proportion of Beijing’s average daily GDP in 2014 ($951.0 million). At the same time, the maximum total economic cost of additional mortality caused by PM_2.5_ per day reached up to $1.085 million during the APEC, which took up 0.11% of Beijing’s average daily GDP. Without intervention measures, the max daily economic cost of additional non-accidental deaths caused by PM_2.5_ could account for 0.29% and 0.33% of Beijing’s average daily GDP. In other words, low PM_2.5_ concentration can significantly reduce the risk of death, which resulted in approximately 0.2% of Beijing’s average daily GDP saved per day. 

With regard to the economic cost of additional non-accidental deaths caused by PM_10_, compared with pre-APEC, the average minimum daily economic loss came down by 50.3% during the APEC, and increased by 26.5% post-APEC. Furthermore, the minimum daily economic loss of additional non-accidental mortality caused by PM_10_ could make up 0.17%, 0.083% and 0.21% of Beijing’s average daily GDP pre-APEC, during the APEC and post-APEC, respectively. The maximum daily economic loss of additional mortality caused by PM_10_ could even take up 0.42% of Beijing’s average daily GDP. The economic cost of cardiovascular diseases deaths and respiratory diseases deaths and proportion of Beijing’s average daily GDP were also presented in Table 4.

## 4. Discussion

This study found that emission control measures made great contributions in reducing the concentrations of PM_2.5_ and PM_10_. The control measures were effective in reducing the number of deaths attributed to particulate matter, and the corresponding health economic cost could be reduced to a certain degree. 

### 4.1. Reduction of Particulate Matters’ Concentrations of Emission Control Measures

As is well known, emission control measures were also implemented during the Olympic Games. In 2008, when the 29th Summer Olympic Games held in Beijing, many scientists evaluated the air quality of Beijing and assessed the effects of emission control measures [16,17,18,19].

Wang and Xie found that on-road air quality was improved effectively due to the 32.3% traffic flow reduction. The average reduction rate of PM_10_ was 28.0% [17]. In our study, the average reduction rate of PM_2.5_ and PM_10_ was 57.0% and 50.6%, respectively. The effects of traffic control measures on APEC days were more obvious compared with Olympic days relatively. Wang et al. found that the concentrations of particulate matter had risen significantly after the Games [19], and Chen et al. also found that air quality improvement during the Olympic Games was real but temporary [20]; our results were consistent with theirs. 

Apart from Beijing, during the period of the Asian Games, Guangzhou government also carried out a series of efficient emission control measures. Results published by Liu et al. showed that the Asian Games abatement strategy reduced emissions of PM_10_ and PM_2.5_ significantly by 26.5% and 23.8% respectively [21].

As for emission control measures carried out during APEC, other research also presented consistent results. Secondary inorganic aerosols (SIA) were reduced by 61%–67% and 51%–57%, and secondary organic aerosols (SOA) came down by 55% and 37%, at 260 m and ground level during the APEC [22]. Liang also found that the 2014 average PM_2.5_ concentration in the first period of 3–12 November was significantly less than the averages in 2013 [23]. Lin et al. concluded that daily average concentration decreased from 98.57 μg/m^3^ to 47.53 μg/m^3^ during “APEC Blue” [24]. As for different types of emission control measures, Davis has found that the driving restrictions that ban most drivers from their vehicles one week day per week on the basis of the last digit of the vehicle’s license plate in Mexico hadn’t improved the air quality because the restriction led to an increase in the total number of vehicles in circulation [25]. However, in Beijing, Viard and Fu have found that every other day driving restrictions based on license plates reduced particulate matter by 18% and one day a week restrictions by 21% [26]; more stringent regulatory efforts on vehicle license plate in Beijing may account for this difference. As for emission control measures carried out in APEC, Wang et al. found that controls are very effective in reducing particulate matter concentrations, and industry source control is the most effective, followed by second aerosol and biomass control, while traffic exhaust control is moderately effective, and the least effective control is for the residue oil combustion source [27]. 

Emission control measures reduced the concentrations of particulate matter to a certain degree controlling for meteorological factors. Regardless of effect size, the emission control measures were indeed effective in alleviating air pollution and improving short-term air quality. The quick recovery of particulate matter concentrations after the emission control measures suggested more effort is needed in China to improve the air quality in the long term.

### 4.2. Health Benefits of Emission Control Measures 

Particulate matter pollution could result in great harm to human health, and consequently the disease burden is enormous. We analyzed additional mortality caused by PM_2.5_ and PM_10_ in different periods of APEC, and results showed that the number of non-accidental cardiovascular and respiratory deaths during APEC-days all dropped approximately 60% compared with non-APEC days, which indicated the effectiveness of air pollution control during the APEC. In our study, PM_2.5_ pollution could cause approximately 13 non-accidental deaths per day without emission control measures on non-APEC days; nevertheless, extreme PM_2.5_ pollution could have caused an average daily 22 premature deaths during the 2013 severe haze event in Beijing [28]. Similar emission control measures were also found effective in alleviate diseases burden. The results of one study conducted during the 2008 Olympic Games [29] showed that the average daily individual mortality during the Game came down by 38.3% and 15.6% respectively compared with pre-Olympic Games and post-Olympic Games. The health risks in post-Olympic Games period were also smaller in contrast to pre-Olympics while our results indicated the health risks post emission control period were more severe; heating supplied in the post-APEC period could be possible account for this difference partly. In general, it seems that the health effects were much greater than in the previous study [29] of interventions during the Olympics. In our opinion, there are several reasons that account for this difference. Firstly, the concentrations of particulate matter in APEC were much higher compared with the Olympics. As is well known, the Olympics were held in summer, while the APEC was held in autumn and winter. Temperature inversion is more likely to form in autumn and winter rather than summer, leading to the accumulation of particulate matter that is harder to diffuse. Apart from that, the greater precipitation in summer is beneficial for particulate matter to spread and eliminate, and heating in winter and incomplete combustion in automobile engine could increase a lot in cold temperature, resulting the growth of particulate matter or the precursors of particulate matter. Given the information mentioned above, the change amount of concentrations of particulate matter is higher in APEC compared with the Olympics. Secondly, the exposed people increased from 2008 to 2014, which means the increase number of potential deaths. To sum up, with the higher amount of concentration change of particulate matter and more exposed people, greater health effects would take place in APEC compare with Olympics. And when taking the ever-increasing medical cost into account, greater economic effects could be achieved in APEC.

### 4.3. Economic Cost Reduction of Emission Control Measures

The economic cost of additional mortality caused by PM_2.5_ and PM_10_ on APEC days decreased substantially compared with non-APEC days. The medical expense of additional mortality saved due to improved air quality could occupy around 0.2% of Beijing’s average daily GDP. According to our research, with emission control measures carried out throughout one year, approximately 3175 deaths induced by PM_2.5_ and 3416 deaths related to PM_10_ could be avoided and a large amount of medical expense could be saved consequently. The medical expense would be gigantic and immeasurable if the air pollution deteriorates and no persistent emission control measures are carried out. According to the results of economic losses evaluation, the haze in January 2013 resulted in 253.8 (95% CI: (170.2, 231.2)) million U.S. $ losses, amounting to 0.08% (95% CI: (0.05%, 0.1%)) of the total 2013 annual GDP of Beijing [28].

### 4.4. The Limitations of This Study

Although we have estimated the efficiency of emission control measures on particulate matter related with health impacts and economic cost during the 2014 APEC with proper methods, some limitations still exist that we cannot ignore. First, due to the variety of health endpoints, we could not analyze all of them, instead we only studied non-accidental, cardiovascular and respiratory diseases which were the dominant causes of death. In this way, our study could not reflect the health benefits of emission control measures completely. Second, we did not take the change of population size and residents’ behaviors into consideration. A previous study showed that pollution sensitive individuals might refrain from going outside on high pollution days (pre-APEC) [30]. In addition, the regulations during the APEC may have limited the size of the population in the city. We only included permanent resident population into our study, while travelers or other floating populations were not taken into account. Our study was in accordance with previous studies conducted during 2008 Beijing Olympic Games or during the periods of APEC Blue and Parade Blue or even the 2013 severe haze event in Beijing, which did not estimate the adaptations or changes in population either, considering the relevant data were hard to obtain [24,28,29]. We think it would be better if the influence of this situation could be explored and taken into consideration in the future studies. Third, previous studies used annual mortality to estimate deaths attributed to particulate matters due to the limited data available [24,29]. In our study, we also used annual mortality to compute daily mortality counts. However, we think that daily mortality was more accurate than annual mortality. Given the short-term changes in exposure are unlikely to have the same effects as long term changes, our results might overstate the efficiency of emission control measures on particulate matter-related health effects. Future studies should be conducted using daily mortality data if available.

## 5. Conclusions

Our study found that the average daily concentrations of PM_2.5_ and PM_10_ during the APEC were reduced compared with the pre-APEC period. However, the concentrations of particulate matter “rebounded” after APEC. Additional mortality caused by PM_2.5_ and PM_10_ during the APEC were the least among three different periods. Consequently, the economic cost associated with mortality caused by PM_2.5_ and PM_10_ reduced during the APEC. 

All the findings suggested that the emission control measures were effective in alleviating air pollution and reducing the health risk and medical expense in Beijing during the 2014 APEC period, but more efforts and appropriate emission control measures are needed for long term and continuous air quality improvement and health protection.

## Figures and Tables

**Table 1:** Characteristics and comparison of particulate matter in various periods.

Air Pollutant	Periods (SAMPLING Days) ^a^	Minimum	P25	Median	P75	Maximum	Mean ± SD	χ^2^	*p* ^b^
PM_2.5_ (μg/m^3^)	Pre-APEC (*n* = 13)	36.43	59.22	104.05	191.41	328.47	130.15 ± 87.18	10.193	0.006
	During-APEC (*n* = 10)	17.67	32.54	54.26	74.12	114.57	55.91 ± 29.33		
	Post-APEC (*n* = 13)	37.27	76.53	90.26	228.38	320.10	145.42 ± 89.58		
PM_10_ (μg/m^3^)	Pre-APEC (*n* = 13)	60.08	87.98	164.64	208.03	339.59	162.16 ± 81.85	13.391	0.001
	During-APEC (*n* = 10)	30.47	57.54	76.61	95.22	157.16	80.05 ± 35.11		
	Post-APEC (*n* = 13)	94.98	122.20	165.18	308.88	371.98	207.69 ± 97.43		

**^a^** We have recorded concentrations of particulate matter on 17 monitoring stations on every sampling day; **^b^** *p* < 0.05.

**Table 2:** The influence of the emission control measures on particulate matter controlling for confounding factors.

Air Pollutants	Parameter	Model Estimate Coefficient	Standard Error	*t*	*p*
PM_2.5_					
	Periods				
	Pre-APEC	50.84	32.16	1.59	0.1144
	Post-APEC	107.73	30.33	3.55	0.0004 **^a^**
	During-APEC (reference)				
PM_10_					
	Periods				
	Pre-APEC	73.19	32.37	2.26	0.0242 **^a^**
	Post-APEC	123.53	32.83	3.76	0.0002 **^a^**
	During-APEC (reference)				

**^a^** *p* < 0.05; Confounding factors include temperature, relative humidity, wind speed, and other confounding factors the monitoring date, hours of the day.

**Table 3:** Estimated daily numbers of deaths attributed to PM pollution pre-, during- and post-APEC.

Health Endpoints	Numbers of Attributable Cases (95% CI)		
	Pre-APEC	Dur-APEC	Post-APEC
PM_2.5_			
Non-accidental deaths	12.9 **^a^**	5.0	14.5
	(10.6, 15.3)	(4.1, 5.9)	(12.4, 17.1)
Cardiovascular disease deaths	4.8	1.8	5.3
	(3.6, 5.8)	(1.4, 2.3)	(4.0, 6.5)
Respiratory disease deaths	2.0	0.8	2.3
	(1.2, 2.9)	(0.5, 1.1)	(1.4, 3.2)
PM_10_			
Non-accidental deaths	14.7	7.3	18.6
	(12.9, 16.0)	(6.4, 8.0)	(16.4, 20.3)
Cardiovascular disease deaths	6.2	3.1	7.9
	(5.4, 7.1)	(2.7, 3.6)	(6.8, 8.9)
Respiratory disease deaths	1.7	0.9	2.2
	(1.2, 2.1)	(0.6, 1.1)	(1.6, 2.7)

**^a^** mean number and the 95% confidence interval were presented.

**Table 4:** Economic cost associated with PM health effects and the corresponding GDP percentage pre, during and post APEC (U.S. $).

Health Endpoints	Economic Cost and GDP Percentage	Pre-APEC	Dur-APEC	Post-APEC
PM_2.5_ non-accidental deaths	Economic cost (min)	1,400,713 (1,150,973–1,661,311) **^a^**	541,912 (445,188–640,636)	1,574,445 (1,346,422–1,856,759)
	Economic cost (max)	2,799,326 (2,300,221–3,320,131)	1,085,010 (889,708–1,280,312)	3,146,529 (2,690,825–3,710,734)
	GDP Percentage	0.15%–0.29%	0.057%–0.11%	0.17%–0.33%
Cardiovascular disease deaths	Economic cost (min)	521,196 (390,897–629,778)	195,448 (152,015–249,740)	575,487 (434,330–705,786)
	Economic cost (max)	1,041,610 (781,207–1,258,612)	390,604 (303,803–499,105)	1,150,111 (868,008–1,410,513)
	GDP Percentage	0.055%–0.11%	0.021%–0.041%	0.061%–0.12%
Respiratory disease deaths	Economic cost (min)	217,165 (130,299–314,889)	86,866 (54,291–119,441)	249,740 (152,015–347,464)
	Economic cost (max)	434,004 (260,402–629,306)	173,602 (108,501–238,702)	499,105 (303,803–694,406)
	GDP Percentage	0.023%–0.046%	0.0091%–0.018%	0.026%–0.052%
PM_10_ non-accidental deaths	Economic cost (min)	1,596,161 (1,400,713–1,737,318)	792,652 (694,927–868,659)	2,019,632 (1,780,751–2,204,223)
	Economic cost (max)	3,189,929 (2,799,326–3,472,032)	1,584,115 (1,388,881–1,736,016)	4,036,237 (3,558,833–4,405,141)
	GDP Percentage	0.17%–0.34%	0.083%–0.17%	0.21%–0.42%
Cardiovascular disease deaths	Economic cost (min)	673,211 (586,345–770,935)	336,605 (293,173–390,897)	857,801 (738,360–966,383)
	Economic cost (max)	1,345,412 (1,171,811–1,540,714)	672,706 (585,905–781,207)	1,714,316 (1,475,614–1,931,318)
	GDP Percentage	0.071%–0.15%	0.035%–0.071%	0.090%–0.18%
Respiratory disease deaths	Economic cost (min)	184,590 (130,299–228,023)	97,724 (65,149–119,441)	238,881 (173,732–293,173)
	Economic cost (max)	368,903 (260,402–455,704)	195,302 (130,201–238,702)	477,404 (347,203–585,905)
	GDP Percentage	0.019%–0.039%	0.010%–0.021%	0.025%–0.050%

**^a^** mean economic cost and the 95% confidence interval were presented.

## Supplementary Materials

The following are available online at www.mdpi.com/1660-4601/14/1/19/s1, Table S1: Characteristics (mean ± SD) and comparison of particulate matter in various monitoring sites, Table S2: Estimated proportion and numbers of deaths attributed to PM pollution pre, during and post APEC using by AirQ+ software.

## Abbreviations

APEC	Asia-Pacific Economic Cooperation
PM	particulate matter
PM_2.5_	particulate matter ≤2.5 μm in aerodynamic diameter, fine particle
PM_10_	particulate matter ≤10 μm in aerodynamic diameter, inhalable particle
DALYs	disability-adjusted life-years
SIA	secondary inorganic aerosol
GDP	gross domestic product
SOA	secondary organic aerosol
