# Supplementary material for: Efficiency of Emission Control Measures on Particulate Matter-Related Health Impacts and Economic Cost during the 2014 Asia-Pacific Economic Cooperation Meeting in Beijing
Source: Int J Environ Res Public Health. 2016 Dec 28;14(1):19. doi: 10.3390/ijerph14010019 (PMC5295270; doi:10.3390/ijerph14010019)
Supplement: Supplementary file 1 [file ijerph-14-00019-s001.pdf]

Supplemental Materials: Efficiency of Emission Control Measures on Particulate Matter Related Health Impacts and Economic Cost during the 2014 Asia-Pacific Economic Cooperation Meeting in Beijing

Qichen Liu, Jing Huang, Bin Guo and Xinbiao Guo

Table S1. Characteristics (mean ± SD) and comparison of particulate matter in various monitoring sites.

Air Pollutants, Various Periods	Urban Sites $N = 8$ [a]	Suburban Sites $N = 3$ [a]	Transportation Sites $N = 5$ [a]	Control Site $N = 1$ [a]	F	p
$PM_{2.5}$ (μg/m³)						
Total	113.11 ± 83.83	95.84 ± 70.69	136.88 ± 96.15	99.19 ± 85.32	1.754	0.159
Pre-APEC	123.77 ± 83.66	113.27 ± 76.17	157.36 ± 99.33	123.09 ± 93.60	0.607	0.613
During-APEC	50.56 ± 33.08	43.74 ± 28.76	75.73 ± 26.41	36.08 ± 25.24	3.628	0.022 [b]
Post-APEC	150.56 ± 88.17	118.48 ± 70.76	163.45 ± 110.62	122.83 ± 86.01	0.756	0.524
PM_{10} (μg/m³)						
Total	162.28 ± 96.89	139.48 ± 81.42	161.42 ± 112.85	113.67 ± 87.48	2.038	0.111
Pre-APEC	170.04 ± 85.13	152.02 ± 76.91	142.10 ± 77.84	124.36 ± 84.83	0.689	0.564
During-APEC	79.78 ± 37.14	71.80 ± 33.27	94.04 ± 40.69	49.56 ± 24.37	2.919	0.047 [b]
Post-APEC	217.97 ± 100.02	178.98 ± 83.14	231.09 ± 140.98	153.12 ± 97.04	1.440	0.243

[a]: representing the total number of monitoring sites of each types; [b]: $p < 0.05$.

Table S2. Estimated proportion and numbers of deaths attributed to PM pollution pre, during and post APEC using by AirQ+ software.

**Health Endpoints**	**Attributed Proportion (95% CI)**			**Attributed Cases (95% CI)**			**Attributed Cases per 100,000 Population at Risk (95% CI)**		
	**Pre-APEC**	**During-APEC**	**Post-APEC**	**Pre-APEC**	**During-APEC**	**Post-APEC**	**Pre-APEC**	**During-APEC**	**Post-APEC**
$PM_{2.5}$									
Non-accidental deaths	4.45% [a] (3.65%, 5.25%)	1.73% (1.41%, 2.04%)	5.01% (4.1%, 5.90%)	12.9 (10.6, 15.2)	5.0 (4.1, 5.9)	14.5 (11.9, 17.1)	21.92 (17.96, 25.84)	8.49 (6.94, 10.04)	24.63 (20.20, 29.02)
Cardiovascular disease deaths	5.14% (3.88%, 6.27%)	2.00% (1.50%, 2.44%)	5.77% (4.36%, 7.03%)	4.7 (3.6, 5.8)	1.8 (1.4, 2.3)	5.3 (4.0, 6.5)	8.05 (6.08, 9.81)	3.12 (2.35, 3.82)	9.04 (6.83, 11.01)
Respiratory disease deaths	5.93% (3.54%, 8.37%)	2.31% (1.37%, 3.28%)	6.66% (3.98%, 9.38%)	2.0 (1.2, 2.9)	0.8 (0.5, 1.1)	2.3 (1.4, 3.2)	3.43 (2.05, 4.85)	1.34 (0.79, 1.90)	3.86 (2.30, 5.43)
PM_{10}									
Non-accidental deaths	4.44% (3.90%, 4.85%)	1.90% (1.67%, 2.08%)	5.82% (5.11%, 6.35%)	12.9 (11.3, 14.1)	5.5 (4.8, 6.0)	16.9 (14.8, 18.4)	21.85 (19.17, 23.84)	9.35 (8.19, 10.22)	28.64 (25.15, 31.23)
Cardiovascular disease deaths	5.92% (5.11%, 6.71%)	2.54% (2.19%, 2.89%)	7.74% (6.70%, 8.77%)	5.5 (4.7, 6.2)	2.3 (2.0, 2.7)	7.1 (6.2, 8.1)	9.27 (8.01, 10.51)	3.98 (3.43, 4.53)	12.12 (10.49, 13.73)
Respiratory disease deaths	4.44% (3.21%, 5.52%)	1.90% (1.37%, 2.37%)	5.82% (4.22%, 7.22%)	1.5 (1.1, 1.9)	0.6 (0.5, 0.8)	2.0 (1.4, 2.5)	2.57 (1.86, 3.20)	1.10 (0.79, 1.37)	3.37 (2.44, 4.18)

[a]: mean number and the 95% confidence interval were presented.

© 2016 by the authors; licensee MDPI, Basel, Switzerland. This article is an open access article distributed under the terms and conditions of the Creative Commons by Attribution (CC-BY) license (http://creativecommons.org/licenses/by/4.0/).
